# 早期非小细胞肺癌ALK阳性患者的临床研究进展

**DOI:** 10.3779/j.issn.1009-3419.2017.02.07

**Published:** 2017-02-20

**Authors:** 琼琼 高, 湘俐 蒋, 纯 黄

**Affiliations:** 300060 天津，天津医科大学肿瘤医院肺部肿瘤内科，国家肿瘤临床医学研究中心，天津市肿瘤防治重点实验室，天津市恶性肿瘤临床医学研究中心 Department of Thoracic Oncology, Tianjin Medical University Cancer Institute and Hospital, National Clinical Research Center for Cancer, Tianjin Key Laboratory of Cancer Prevention and Therapy, Tianjin's Clinical Research Center for Cancer, Tianjin 300060, China

**Keywords:** 肺肿瘤, ALK, 检测, 临床病理特征, 预后, Lung neoplasms, ALK, Testing, Clinicopathological implication, Outcome

## Abstract

肺癌是我国癌症死亡最主要的原因。其中非小细胞肺癌（non-small cell lung cancer, NSCLC）占肺癌患者的85%，且大部分患者初诊时即为晚期。针对晚期NSCLC患者，分子靶向治疗成为人们关注的热点。棘皮动物微管相关蛋白-间变淋巴瘤激酶（echinoderm microtubule-associated protein-like 4 gene and the anaplastic lymphoma kinase gene, *EML4*-*ALK*）是NSCLC最常见的分子靶点之一，其特异性的小分子酪氨酸激酶抑制剂（tyrosine kinase inhibitors, TKIs）已被批准应用于ALK阳性晚期NSCLC患者的治疗。然而，*ALK*融合基因对早期NSCLC患者预后的影响，以及ALK阳性的早期NSCLC患者应用TKIs的必要性等问题尚不明确。本文主要围绕ALK阳性NSCLC患者的检测进展，早期ALK阳性NSCLC患者的临床病理特征、预后、ALK-TKIs应用必要性等情况做一简要综述。

## Abstract (en)

Lung cancer is the leading cause of cancer death in China. Non-small cell lung cancer (NSCLC) accounts for 85% of lung cancer cases, with the majority of the cases diagnosed at the advanced stage. Molecular targeted therapy is becoming the focus attention for advanced NSCLC. Echinoderm microtubule-associated protein-like 4 gene and the anaplastic lymphoma kinase gene (EML4-ALK) is among the most common molecular targets of NSCLC; its specific small-molecule tyrosine kinase inhibitors (TKIs) are approved for use in advanced NSCLC cases of ALK-positive. However, the influence of *EML4*-*ALK* fusion gene on the outcome of early-stage NSCLC cases and the necessity of application of TKIs for early-stage ALK-positive NSCLC patients are still uncertain. In this paper, we summarized the progression of testing methods for ALKpositive NSCLC patients as well as clinicopathological implication, outcome, and necessity of application of TKIs for earlystage ALK-positive NSCLC patients.

肺癌是我国最常见的恶性肿瘤之一，近年来成为我国癌症死亡最主要的原因^[1]^。据国家癌症中心2015年发布的数据显示：2006年-2011年我国肺癌5年患病率是130.2（1/10万）。其中男性84.6（1/10万），居恶性肿瘤第2位；女性45.6（1/10万），居恶性肿瘤第4位^[2]^。其中非小细胞肺癌（non-small cell lung cancer, NSCLC）占肺癌患者的85%^[3]^。流行病学数据显示，不足43%的NSCLC患者初诊时为早期，并获得根治性治疗机会。然而，57%以上的NSCLC患者初诊时即为晚期，已经出现远处转移。对于晚期NSCLC患者，除一线含铂双药化疗外，分子靶向治疗越来越多的受到人们的关注。棘皮动物微管相关蛋白-间变淋巴瘤激酶（echinoderm microtubule-associated protein-like 4 gene and the anaplastic lymphoma kinase gene, EML4-ALK）等分子亚型的出现，使得人们对NSCLC的发生、发展的分子机制进一步明确，而针对EML4-ALK致癌靶点的小分子酪氨酸激酶抑制剂（tyrosine kinase inhibitors, TKIs）-克唑替尼（crizotinib）等，因其具备良好的疗效和安全性，已被批准应用于ALK阳性晚期NSCLC患者的治疗。对于ALK阳性早期NSCLC患者，ALK-TKIs应用必要性尚不明确，本文主要介绍ALK阳性NSCLC患者的检测进展，ALK阳性早期NSCLC患者的临床病理特征、预后，评价ALK-TKIs在ALK阳性早期NSCLC患者中的地位和意义。

## 1 *EML4*-*ALK*融合基因的发现

EML4-ALK是在2007年由Soda等^[4]^通过应用酪氨酸激酶蛋白组学技术从一个肺腺癌患者肿瘤组织中筛选致癌基因时首次发现的。EML4属于棘皮动物微管蛋白相关类蛋白家族，由N端Basic区、HELP域和WD重复区构成；ALK属于胰岛素受体超家族，由细胞外配体结合区、跨膜区及胞内的酪氨酸激酶区组成。ALK蛋白通过活化下游的STAT3和MARK信号传导通路及激活RAS/ERK、PI3K/AKT等多条其他的信号通路来调控细胞的增殖和凋亡。正是由于2号染色体短臂的微小倒置导致EML4 N端Basic区、HELP域和部分WD重复区在ALK胞内的酪氨酸激酶区发生融合，形成*EML4*-*ALK*融合基因。融合基因的EML4部分均具有致癌活性，其中以Basic区致癌活性最高，这种致癌活性依赖于融合伴侣EML4和ALK的二聚作用对酪氨酸激酶的激活。EML4-ALK在体内及体外均拥有强大的致癌活性^[4, 5]^，这种致癌活性可以被针对ALK靶点的小分子TKIs有效阻断^[5, 6]^，这为EML4-ALK作为肺癌发生的关键驱动因子提供了证据。EML4-ALK存在十余种融合基因亚型，最常见的融合亚型为E13:A20和E6a/b:A20，发生率分别为33%和29%^[7, 8]^。当然，在NSCLC中，除了EML4作为ALK的常见融合伴侣外，研究还相继发现了其他的ALK融合伴侣，包括TFG^[9]^、KIF5B^[10]^、KLC1^[11]^、HIP1^[12]^、TPR^[13]^、SEC31A^[14]^等。

## 2 *ALK*融合基因检测技术的发展

现在有3种不同的方式来检测*ALK*融合基因的表达情况，分别是免疫组化（immunohistochemistry, IHC）、荧光原位杂交（fluorescence *in situ* hybridization, FISH）及逆转录聚合酶链反应（reverse transcriptase-polymerase chain reaction, RT-PCR）。在临床上，FISH和RT-PCR技术检测*ALK*融合基因已有相应试剂盒通过美国食品药品监督管理局（Food and Drug Administration, FDA）及国家食品药品监督管理总局（China Food and Drug Administration, CFDA）的认证。2013年美国国家综合癌症网（National Comprehensive Cancer Network, NCCN）指南也明确表示FISH可作为*ALK*融合基因检测的金标准。然而，FISH荧光信号消减迅速，难以测定肿瘤整体形态学及肿瘤异质性，且价格昂贵^[15]^；RT-PCR对引物要求较高，需要较多RNA及石蜡组织样本易降解^[16]^等因素，使得FISH和RT-PCR技术不易普及。相比而言，IHC便宜、高效、快速，因而被广泛应用于常规的病理学实验室。然而，IHC的实验操作和结果评价存在一定的主观性，且IHC检测质量很大程度上取决于抗体的质量，但目前已开发的商业化EML4-ALK抗体的特异性及敏感性不够高，低表达的EML4-ALK融合蛋白往往被漏检^[17]^。所以开发高特异性及高敏感性的抗体至关重要。Pyo等^[18]^在IHC筛查ALK重排的系统回顾和*meta*分析中指出：IHC的敏感性和特异性分别为92%和91%。刘畅等^[19]^在免疫组化法检测*EML4*-*ALK*融合突变价值的*meta*分析中得出特异性抗体IHC法检测*EML4*-*ALK*融合基因的方法可行，具有高特异度和敏感度，可作为一种简单快速的筛查方法，具有临床应用价值。而针对4种不同的ALK抗体D5F3（ventana）、D5F3（CST）、1A4/1H7（OriGene Tech）和5A4（Abcam）其敏感性分别为93.8%、84.4%、93.8%和56.3%，其特异性和阳性预测值均为100%^[20]^。尤其是新研发的单克隆抗体D5F3（ventana）大大提高了检测的特异性和敏感性^[20, 21]^，甚至有研究^[22]^认为其可以作为有明确染色模式下的*ALK*融合基因的独立检测手段。最近，罗氏公司推出的用于识别肺癌患者EML4-ALK融合蛋白表达的首款全自动VENTANA ALK（D5F3）IHC检测试剂盒获得FDA及CFDA认证，研究表明，该方法检测EML4-ALK融合蛋白的特异性和敏感性分别为98%和100%^[23]^，金贻铎等^[24]^研究指出相较于RT-PCR，手工IHC检测EML4-ALK融合蛋白的敏感性、特异性、阳性预测值和阴性预测值分别为82.4%、95.1%、73.7%和97%，二者的总体符合率为93.3%，VENTANA IHC检测EML4-ALK融合蛋白的敏感性、特异性、阳性预测值和阴性预测值分别为93.3%、100%、100%和99.1%，二者的总体符合率为99.1%。VENTANA IHC相较于手工IHC具备更高的特异性和敏感性，且该方法已被批准用于筛选适合服用辉瑞制药有限公司的克唑替尼的患者，这极大肯定了IHC在ALK检测中的地位和作用。

## 3 *ALK*融合基因阳性患者的临床病理特征

因检测方法、检测人群不同，*ALK*融合基因在NSCLC患者中的阳性率为1.4%-13%^[25-28]^，且在Ⅳ期出现转移的NSCLC患者中，*ALK*融合基因的阳性率比较高。有研究指出晚期NSCLC患者*ALK*融合基因的阳性率为8.7%-9.0%^[29, 30]^，而在早期NSCLC患者中的阳性率仅为2.4%-8.6%^[31-35]^，明显低于晚期患者。这点和Zhao等^[36]^在关于*ALK*融合基因临床特征的*meta*分析中得出的结论是一致的，他指出*ALK*融合基因在Ⅲ期-Ⅳ期的NSCLC患者中的检出率高于Ⅰ期-Ⅱ期患者，即*ALK*融合基因在晚期NSCLC患者的阳性率高于早期NSCLC患者。关于早期和晚期*ALK*融合基因阳性患者的临床病理特征普遍达成共识，即：年轻、不吸烟或少量吸烟、富含印戒细胞或实体成分的腺癌、*EGFR*或*KRAS*突变野生型的NSCLC^[25-35]^。有研究指出*ALK*融合基因在*EGFR*或*KRAS*突变阴性的NSCLC患者中的阳性率高达25.7%-34%^[25, 32, 37]^。

## 4 *ALK*融合基因对早期NSCLC患者预后的影响

一直以来，关于*ALK*融合基因表达情况是否影响早期NSCLC患者的预后尚存有一些争议。韩国Paik等^[31]^回顾性研究了接受根治性手术治疗的早期NSCLC中ALK阳性患者的临床病理特征和预后情况，研究指出，运用FISH技术对735例患者手术标本进行*ALK*融合基因检测，其中ALK阳性患者28例（3.8%），ALK阳性率在腺癌、女性、不吸烟患者中明显升高，分别为6.8%、7.6%和8.9%；ALK阳性患者中位年龄55岁，明显低于ALK阴性患者（*P* < 0.001）；中位随访时间41.6个月时，ALK阳性患者与ALK阴性患者的总生存（overall survival, OS）分别为97.7个月*vs* 78.9个月（*P*=0.10），无病生存期（disease-free survival, DFS）分别为76.4个月*vs* 71.3个月（*P*=0.66），ALK阳性患者与ALK阴性患者的DFS及OS无统计学差异，*ALK*融合基因并非是影响患者DFS和OS的预后因素。研究还发现ALK阳性患者T分期级别较阴性患者低，且主要集中在T1期（*P*=0.02），但在腺癌患者中淋巴结转移级别较阴性患者高，这说明了ALK阳性患者具备原发肿瘤虽小但易出现早期淋巴结转移的独特的生物学特征，也进一步解释了晚期NSCLC患者ALK阳性率较早期患者高的原因。而来自韩国的另外一项研究^[32]^同样指出，ALK阳性患者的T分期级别较阴性者低，但未指出两组患者淋巴结转移情况的不同。该研究还发现，在纳入研究的162例接受根治性手术治疗的早期不吸烟肺腺癌患者中，ALK阳性患者14例（8.6%）；中位随访时间40.8个月，ALK阳性患者与ALK阴性患者的OS无统计学差异，但ALK阳性患者DFS更短（*P*=0.022），提示ALK阳性患者更易出现术后疾病的复发。泰国Tantraworasin等^[34]^研究发现，在267例接受根治性手术的NSCLC患者中，ALK阳性更易出现在年龄小于55岁、存在脏胸膜受侵犯、有肺癌家族史的NSCLC患者中，且ALK阳性患者更易出现肿瘤的复发；该研究指出*ALK*融合基因并非是影响DFS和OS的预后因素，这一点和Paik等^[31]^的研究结果是一致的。然而韩国Kim等^[26]^研究发现，在119例接受根治性手术治疗的早期NSCLC患者中，ALK阳性患者和ALK阴性患者的RFS无统计学差异，即ALK阳性和肿瘤复发无关；另外该研究还指出ALK阳性患者OS更短，说明ALK阳性可能是早期NSCLC患者的预后不良的因素。张梦雪等^[38]^研究指出：在95例早期NSCLC患者中，ALK阳性患者OS更长，预后更好。Blackhall等^[33]^的关于欧洲人群的研究显示，在1, 281例接受根治性手术切除的肺腺癌患者中，ALK阳性患者较ALK阴性患者OS更长，即ALK阳性患者较ALK阴性患者预后好。

为了进一步分析造成不同结论的原因，有研究者将早期NSCLC按照病理或临床分期分成不同的亚组来研究。Ohba等^[35]^指出，在256例病理分期为Ⅰ期的日本肺腺癌患者中，*ALK*融合基因并非是影响肺腺癌患者DFS和OS的预后因素。Zhou等^[27]^的研究发现，在488例中国NSCLC患者中，165例为Ⅲa期，仅在Ⅲa期NSCLC患者中，ALK阳性者较ALK阴性者DFS更短，其他分期组ALK阳性和阴性者的DFS无统计学差异。

分析以上资料，我们发现：ALK阳性对亚裔人群预后的影响各不相同。这种差异很可能是受回顾性研究的限制、随访时间短、样本量较小、检测技术等混杂因素的影响，而比较欧美和亚裔人群的研究结果提示我们ALK阳性对不同种族人群预后的影响可能不同。

*ALK*融合基因对晚期NSCLC患者预后的影响现已比较明确。研究指出ALK阳性患者和ALK阴性患者对含铂化疗方案的敏感性无统计学差异，ALK阳性患者和ALK阴性患者DFS和OS无统计学差异^[25, 30, 39]^。而Yang等^[39]^的研究进一步指出，ALK阳性者较ALK阴性者有着更短的DFS，但OS无统计学差异；ALK阳性肺腺癌患者在被确诊后的5年内，疾病的进展和复发率是ALK阴性患者的2倍。对晚期NSCLC患者而言，*ALK*融合基因并非影响预后的因素，ALK阳性患者更易出现疾病的进展。

## 5 ALK抑制剂

克唑替尼（crizotinib）是2005年合成的一种ATP竞争性TKI，具有抗ALK活性的脱靶效应，可作为小分子ATP模拟化合物与ATP靶位结合，从而阻滞了ALK下游信号通路的激活，进一步阻滞肿瘤细胞生长及侵袭^[40]^。Ⅲ期临床试验PROFILE1014^[41]^结果显示：克唑替尼作为一线用药对于延长ALK阳性晚期NSCLC患者的无进展生存期的疗效要优于标准化疗。尽管ALK阳性晚期NSCLC患者可以从克唑替尼中获益，但与EGFR-TKIs一样，大多数患者在获得8个月-10个月的肿瘤控制后，最终会发展成获得性耐药。耐药机制主要包括ALK激酶区二次突变、*ALK*融合基因扩增、旁路激活途径等。多项研究对解决克唑替尼耐药问题进行了探索，Ou等^[42]^研究表明：服用克唑替尼患者出现疾病进展后继续用药比停止服药获益明显。第2代ALK抑制剂由于其在结构上与克唑替尼差异明显，不仅对克唑替尼敏感的ALK阳性晚期NSCLC患者有效，而且对克唑替尼耐药患者更是表现出强大的疗效。目前经FDA批准上市的有Ceritinib，正在进行临床研究的药物有Alectinib、AP26113。Ceritinib能抑制ALK自身磷酸化和ALK介导下游信号蛋白（STAT3）的磷酸化，Friboulet等^[43]^研究指出：Ceritinib对L1196M、G1269A、S1206Y、I1171T等ALK激酶区突变引起的耐药具有显著活性；Alectinib在体外化合物筛选时具有很强的ALK抑制活性，Kodama等^[44]^研究发现：Alectinib可以对已知的大多数ALK耐药类型有效；AP26113是ALK、EGFR双重抑制剂，对绝大部分ALK耐药疗效显著，Viala等^[45]^发现AP26113等可有效解决L1196M突变引起的耐药问题。另外，HSP90抑制剂可以下调ALK融合蛋白的表达水平，对ALK阳性的未经克唑替尼处理或对克唑替尼耐药的细胞均具有良好的活性，相关研究已进入临床试验阶段^[46]^。

## 6 小结

*EML4*-*ALK*融合基因是肺癌的重要分子亚型，由于2号染色体短臂的微小倒置导致EML4N端Basic区、HELP域和部分WD重复区在ALK胞内的酪氨酸激酶区发生融合，EML4-ALK存在十余种融合基因亚型，除了EML4作为ALK的常见融合伴侣外，研究还相继发现了其他至少6种ALK融合伴侣。综合IHC、FISH和RT-PCR检测技术，IHC的应用更为重要和普及，尤其是全自动VENTANA ALK（D5F3）IHC检测试剂盒的获批，极大肯定了IHC在ALK检测中的地位和作用。*EML4*-*ALK*融合基因多见于年轻、不吸烟或少量吸烟、富含印戒细胞或实性型腺癌、*EGFR*或*KRAS*突变野生型的NSCLC患者，且早期NSCLC患者中*EML4*-*ALK*融合基因的检出率低于晚期患者。克唑替尼等ALK-TKIs在晚期ALK阳性NSCLC患者中的治疗作用已得到广泛认可，但目前缺乏资料证明ALK-TKIs可使早期ALK阳性NSCLC患者受益，这或许是因为*ALK*融合基因对早期NSCLC患者预后的影响尚不明确。本文对有限的早期资料进行了总结，多数研究证明*ALK*融合基因和NSCLC患者的预后无关，仅Kim等^[26]^的研究指出ALK阳性早期NSCLC患者预后不良，为了进一步明确这一问题，我们收集了1, 108例多中心的早期NSCLC患者的标本和临床资料进行分析，目前尚在进行中，期待能得出有临床指导意义的结果。
